# Case Report: Tattoo sarcoidosis with epithelioid cell granuloma positive for *Propionibacterium acne*s

**DOI:** 10.3389/fmed.2025.1552114

**Published:** 2025-03-19

**Authors:** Yuiko Masuda, Hiroko Okabayashi, Kimitaka Akaike, Shohei Hamada, Aiko Masunaga, Hidenori Ichiyasu, Kenichi Ohashi, Takuro Sakagami

**Affiliations:** ^1^Department of Respiratory Medicine, Kumamoto University Hospital, Faculty of Life, Sciences, Kumamoto University, Kumamoto, Japan; ^2^Department of Human Pathology, Institute of Science Tokyo, Tokyo, Japan

**Keywords:** tattoo sarcoidosis, sarcoidosis, tattoo, *Propionibacterium acnes*, *Cutibacterium acnes*

## Abstract

**Introduction:**

Tattoo sarcoidosis is characterized by a granulomatous reaction localized to the tattoo site and typical systemic symptoms of sarcoidosis. Herein, we report the case of a patient diagnosed with tattoo sarcoidosis.

**Case report:**

A 28-year-old man presented with subcutaneous nodules at two tattoo sites, bilateral hilar and mediastinal lymphadenopathy, multiple micronodules predominantly in the upper lobes of both lungs, elevated serum angiotensin-converting enzyme and soluble interleukin-2 receptor levels, hypercalcemia, and renal dysfunction. Skin biopsy of a subcutaneous nodule revealed epithelioid cell granulomas. Although periodic acid-Schiff, Grocott methenamine silver, and acid-fast staining showed negative results, antibody staining for *Propionibacterium acnes* within the epithelioid cell granuloma was positive. Remarkably, all lesions spontaneously resolved, and the systemic manifestations also improved without medical treatment.

**Conclusion:**

The pathogenesis of tattoo sarcoidosis remains unknown, although an immune response to tattoo pigments has been suspected. However, there is a theory that *P. acnes* is the causative agent of sarcoidosis. In the present case, the detection of *P. acnes* within the epithelioid cell granuloma suggests that the bacterium may play a role in the etiology of tattoo sarcoidosis.

## 1 Introduction

Sarcoidosis is a rare, multisystem inflammatory disease characterized by granuloma formation in various tissues and organs. While the exact cause remains unknown, it is believed to result from an abnormal immune response triggered by environmental factors, infectious agents, or genetic predispositions (1). A hypothesis suggests that *Propionibacterium acnes*—currently referred to as *Cutibacterium acnes*—may play a role in its pathogenesis (2).

Tattoos trigger various dermatological conditions, and localized granulomatous reactions at tattoo sites are referred to as tattoo granulomas. Tattoo sarcoidosis describes cases in which granulomatous reactions are accompanied by systemic manifestations characteristic of sarcoidosis. Although the immune response to tattoo pigments has been proposed as a cause of tattoo sarcoidosis, the exact etiology remains unclear (3). Herein, we present a case of tattoo sarcoidosis in which *P. acnes* was detected within an epithelioid cell granuloma.

## 2 Case description

**Figure 1:**
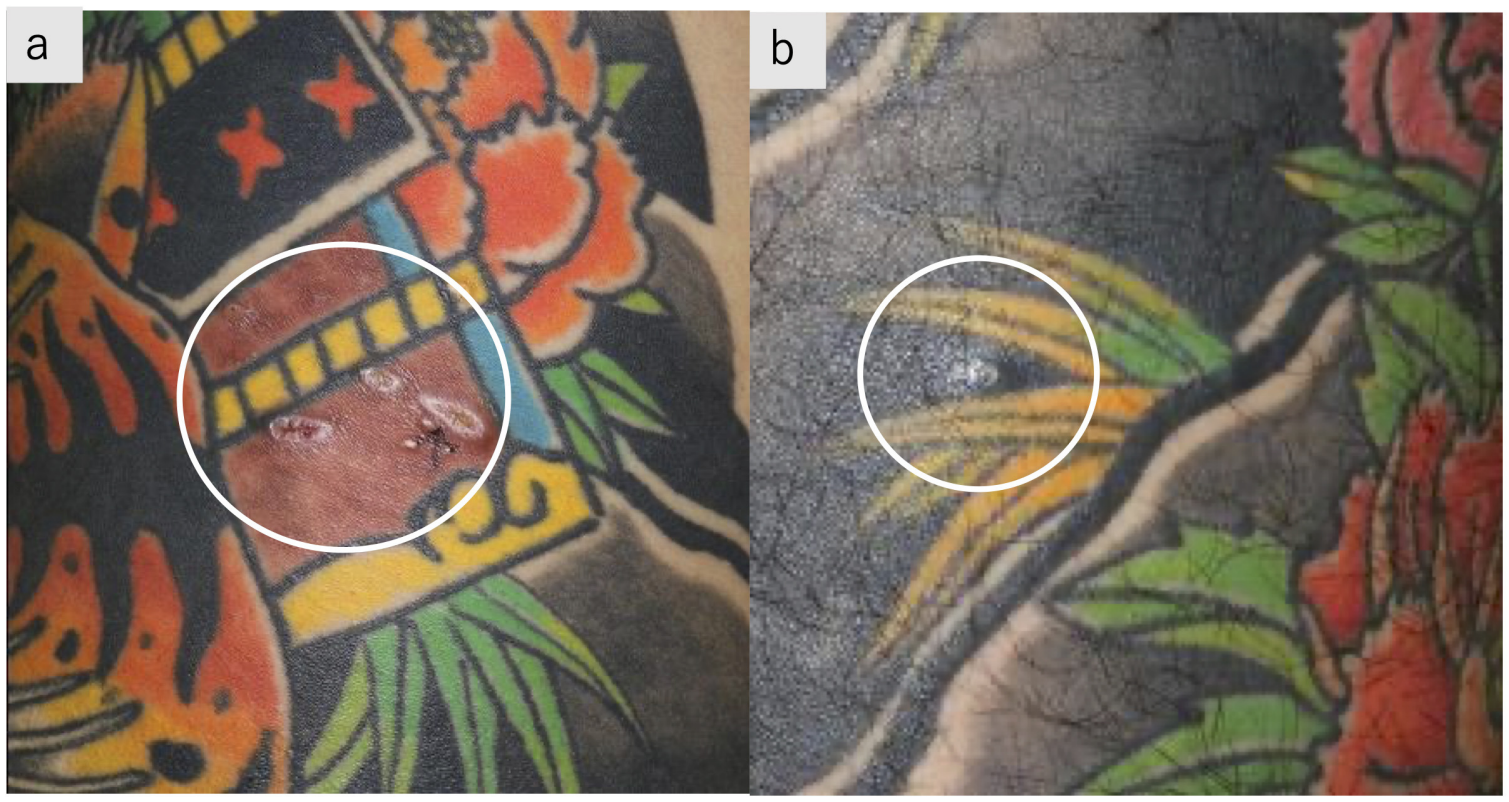
Tattoos with subcutaneous nodules. Subcutaneous nodules palpable on reddish-brown tattoos on the right lower back **(a)** and black-inked tattoos on the left thigh **(b)**.

A 28-year-old man was referred to our hospital after abnormal chest shadows were detected during a routine health examination. He noted subcutaneous nodules in the tattooed areas. He had a 1-year history of chronic cough, dyspnea on exertion, and loss of appetite and weight. He had completed a full-body tattoo over a 4-year period, beginning 5 years prior to presentation. The patient was a current smoker, with an approximately 18 pack-year smoking history. On admission, his temperature was 36.4°C, and oxygen saturation was 97% on room air. Subcutaneous nodules were palpable on the reddish-brown tattoos on the lower back and black-inked tattoos on the left thigh (Figure 1).

**Figure 2:**
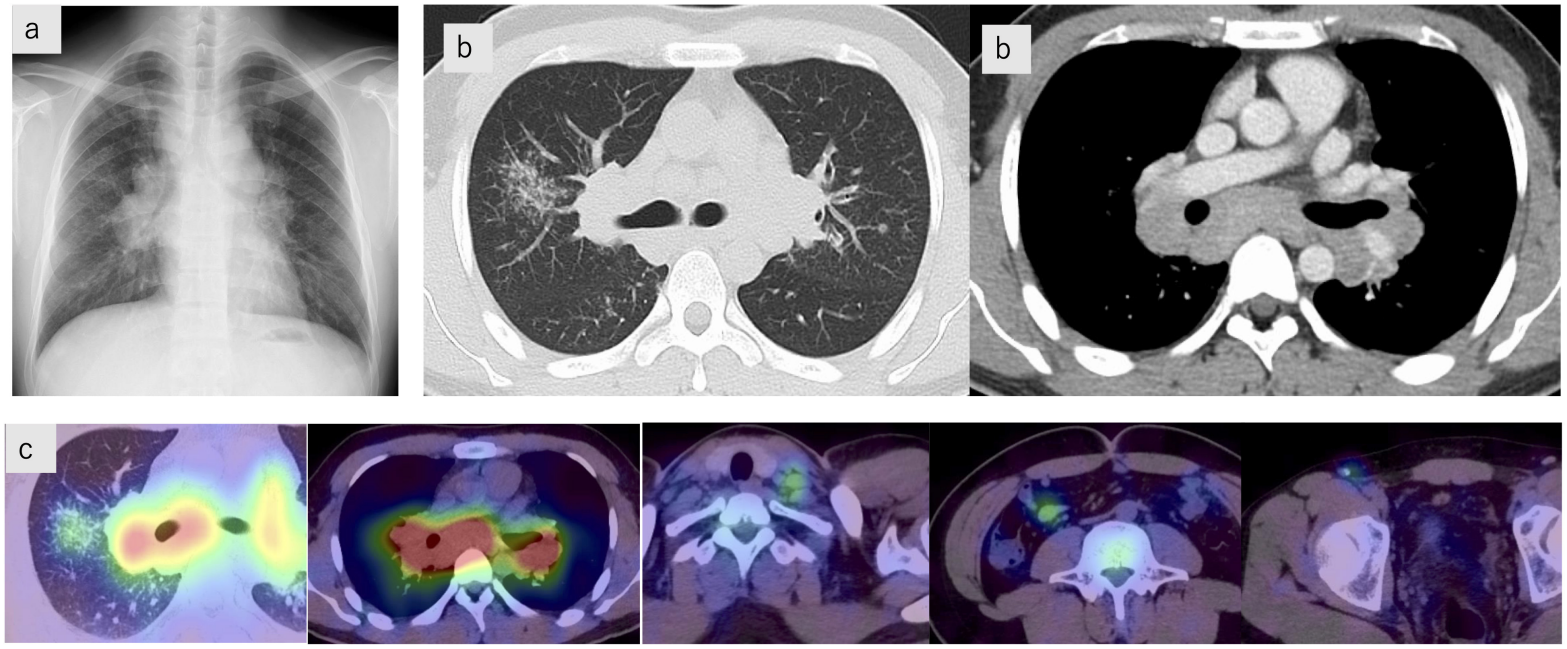
Imaging findings. **(a)** Chest radiograph showing bilateral hilar lymphadenopathy. **(b)** Chest computed tomography scans showing multiple nodular and granular opacities along the bronchovascular bundles, predominantly in the upper lobes, forming a galaxy sign in the right upper lobe. Enlarged hilar and mediastinal lymph nodes are also observed. **(c)** Gallium scintigraphy demonstrates significant uptake in the hilar and mediastinal lymph nodes, the left supraclavicular lymph node, the ileocolic lymph node, the right inguinal lymph node, and the granular shadows of the right upper lobe.

Investigations upon admission showed renal dysfunction, hypercalcemia, and elevated urinary N-acetyl-β-D-glucosaminidase. Serum angiotensin-converting enzyme (ACE), soluble interleukin-2 receptor (sIL-2R), and lysozyme levels were also elevated (Table 1). Chest radiographs showed bilateral hilar lymphadenopathy (Figure 2a), and high-resolution computed tomography (CT) scans revealed multiple nodular and granular opacities along the bronchovascular bundles, predominantly in the upper lobes, forming a galaxy sign in the right upper lobe. Enlarged hilar and mediastinal lymph nodes were observed (Figure 2b). Gallium scintigraphy revealed significant uptake in the hilar and mediastinal lymph nodes, left supraclavicular lymph node, ileocolic lymph node, right inguinal lymph node, and granular shadows of the right upper lobe (Figure 2c). Pulmonary function testing revealed no restrictive impairment (%FVC 86.8%) but did indicate diffusion impairment (%DL_CO_ 73.4%).

**Table 1:** Laboratory findings.

**Parameter**	**Result**	**Reference range**
**Blood test**		
White blood cell (/μl)	5.7 × 103	3.3–8.6 × 103
Neutrophils (/μl)	4.1 × 103	2.4–4.3 × 103
Lymphocytes (/μl)	0.6 × 103	1.0–2.7 × 103
Eosinophils (/μl)	0.3 × 103	0.3–0.5 × 103
Hemoglobin (g/dl)	14.3	13.7–16.8
Platelet count (/μl)	37 × 104	15.8–34.8 × 104
Total protein (g/dl)	7.1	6.6–8.1
Albumin (g/dl)	4.4	4.1–5.1
Na (mEq/L)	139	138–145
K (mEq/L)	4.2	3.6–4.8
Cl (mEq/L)	103	101–108
Ca (mg/dl)	10.3	8.8–10.1
IP (mg/dl)	3.7	2.7–4.6
BUN (mg/dl)	14.5	8.0–20
Creatinine (mg/dl)	1.34	0.65–1.07
AST (U/L)	20	13–30
ALT (U/L)	18	10–42
LDH (U/L)	228	124–222
CRP (mg/dl)	1.26	0.00–0.14
ACE (IU/L)	27.6	8.3–21.4
KL-6 (U/mL)	314	105.3–401.2
sIL-2R (U/mL)	1,836	121–613
Lysozyme (μg/mL)	36.9	5.0–10.2
IgG (mg/dl)	937	861–1,747
IgA (mg/dl)	192	93–393
IgM (mg/dl)	93	33–183
**Urinalysis**		
Protein	Negative	
Blood	Negative	
Glucose	Negative	
White blood cells (/HPF)	1–4	< 1
Red blood cells (/HPF)	< 1	< 1
U-Crea (mg/dl)	322.1	40–260
U-TP (mg/dl)	4.9	< 15
UPCR (g/gCr)	0.02	< 0.15
U-BMG (mg/L)	0.275	< 0.265
U-NAG (IU/L)	14.4	< 11.5

Na, sodium; K, potassium; Cl, chloride; Ca, calcium; IP, inorganic phosphorus; BUN, blood urea nitrogen; AST, aspartate aminotransferase; ALT, alanine aminotransferase; LDH, lactate dehydrogenase; CRP, C-reactive protein; ACE, angiotensin-converting enzyme; KL-6, Krebs von den Lungen-6; sIL-2R, soluble IL-2 receptor; IgG, immunoglobulin G; IgA, immunoglobulin A; IgM, immunoglobulin M; U-Crea, urinary creatinine; U-TP, urinary total protein; UPCR, urine protein-to-creatinine ratio; U-BMG, urinary β2-microglobulin; U-NAG, urinary N-acetyl-β-D-glucosaminidase; HPF, high power field.

**Figure 3:**
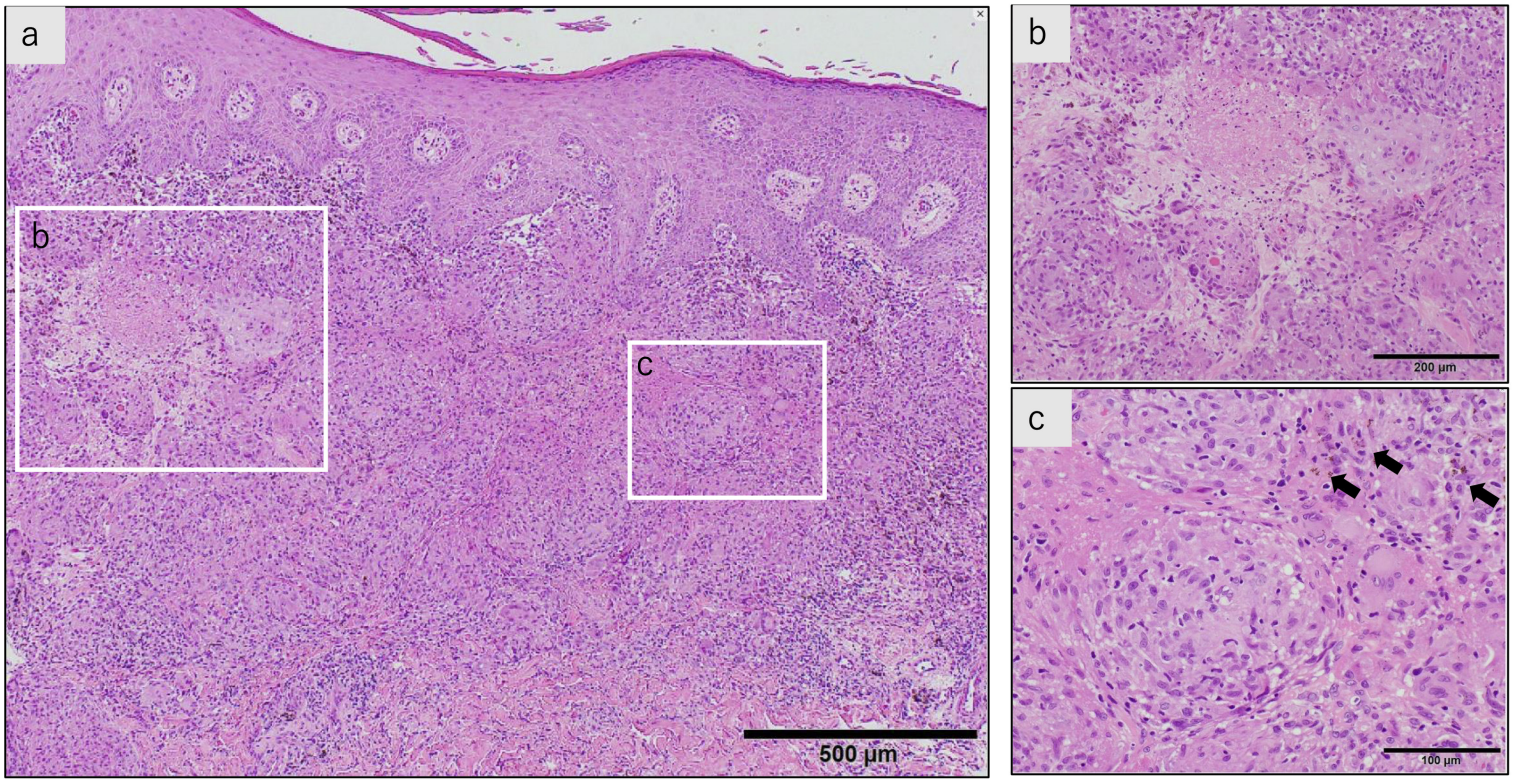
A skin biopsy of the reddish-brown tattoo on the right lower back **(**hematoxylin-eosin stain). **(a)** The stratum corneum shows parakeratosis, and the epidermis is thickened with spongiosis. There is a marked infiltration of inflammatory cells just below the epidermis, and multiple granulomas composed of epithelioid cells and lymphocytes are present. Langerhans giant cells are also seen. **(b)** Fibrinoid necrosis is observed in some areas. **(c)** Within the epithelioid cell granulomas, reddish-brown granular pigments, presumed to be tattoo ink, are observed (arrow).

**Figure 4:**
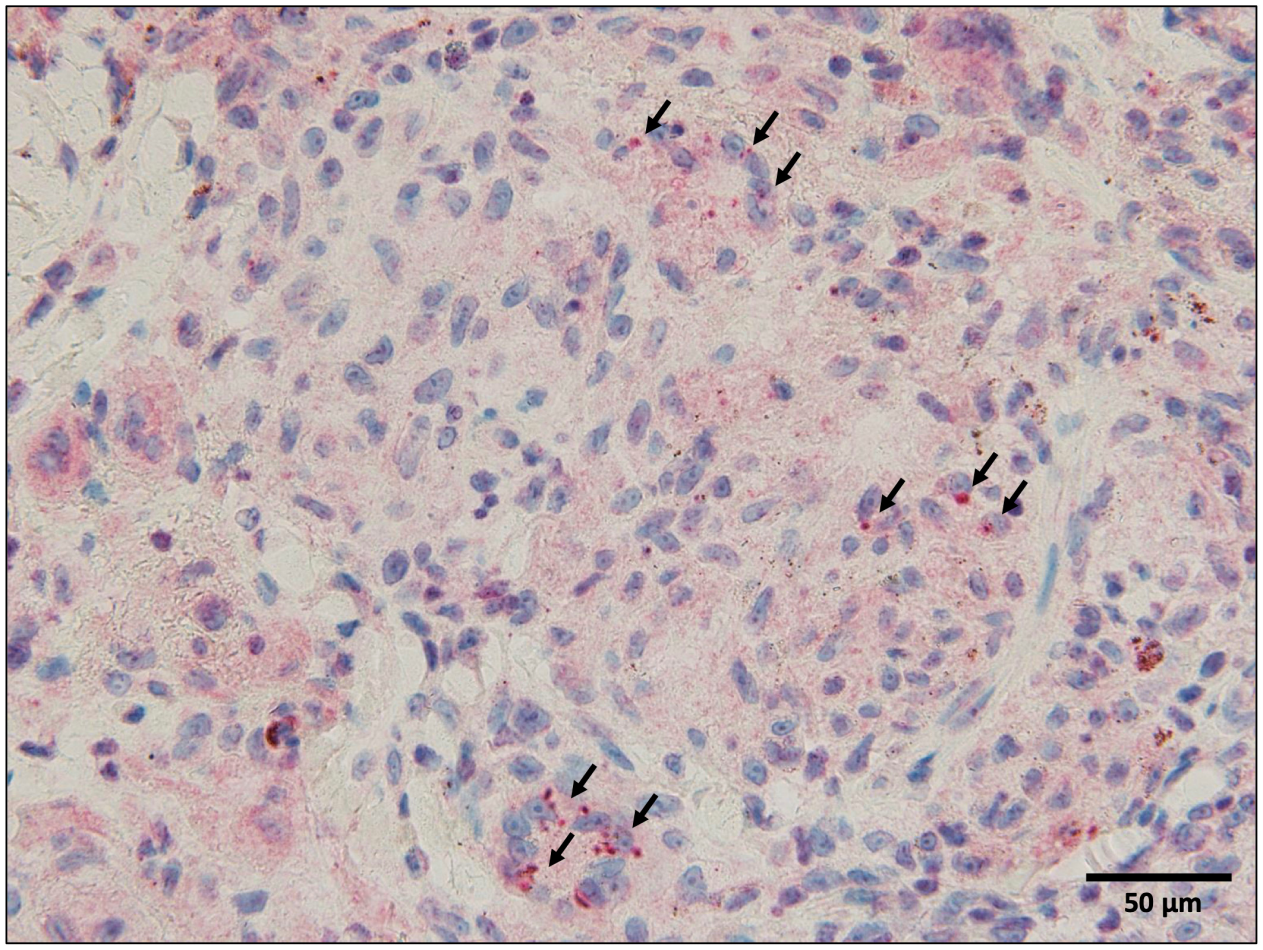
Immunohistochemistry with the *Propionibacterium acnes*-specific monoclonal antibody (PAB antibody) Since the tattoo pigment is reddish-brown, the chromogen 3-amino-9-ethylcarbazole (AEC) was used for immunohistochemical staining to differentiate between positive findings and tattoo pigment, and *P. acnes* is stained red. Immunohistochemistry with the PAB antibody reveals fine red granules (arrows), indicating *P. acnes* within the epithelioid cells, and multinucleated giant cells of the granulomatous lesion.

Bronchoalveolar lavage fluid analysis revealed a lymphocyte ratio of 6.5% and an elevated CD4/CD8 ratio of 7.2. Endobronchial ultrasonography-guided transbronchial needle aspiration was performed on the subcarinal lymph nodes; however, the specimen was of poor quality. Skin biopsy of the reddish-brown tattoo on the right lower back revealed numerous epithelioid cell granulomas in the upper dermis, with some areas of fibrinoid necrosis (Figure 3). Staining for pathogens, such as periodic acid-Schiff, Grocott's methenamine silver, and acid-fast bacilli, was negative. Immunohistochemistry with the *P. acnes*-specific monoclonal antibody (PAB antibody) revealed fine red granules, indicating *P. acnes* within the epithelioid cells and multinucleated giant cells of the granulomatous lesion (Figure 4). Based on these findings, the patient was diagnosed with tattoo-associated sarcoidosis. In this case, an elevated serum creatinine level and an increased urinary NAG/creatinine ratio (44.7 IU/g·Cr) were observed, raising the possibility of interstitial nephritis. However, Ga scintigraphy showed no renal uptake, and renal biopsy could not be performed due to the patient's lack of consent.

Although systemic steroid therapy was planned, the patient's symptoms, renal dysfunction, hypercalcemia, and bilateral hilar lymphadenopathy improved during the investigation period; therefore, pharmacological treatment was deferred. One year later, chest CT revealed reduced hilar and mediastinal lymphadenopathy and pulmonary lesions, and the serum ACE and sIL-2R levels also normalized. Subcutaneous nodules at tattoo sites resolved spontaneously. The patient continues with outpatient follow-up with no signs of recurrence.

## 3 Discussion

Tattoo sarcoidosis is rare, and its incidence and prevalence remain unclear. According to a study by Ohtsuka et al. examining tattoo sarcoidosis in the Japanese population, the condition predominantly affected men and showed a bimodal age distribution, peaking in the 20s−30s and again in the 60s. The median time from tattoo application to onset was 13.7 years. In most cases, tattoo-related reactions were the initial manifestations of sarcoidosis. The possible reasons for the long duration between tattoo application and symptom onset include the slow degradation of tattoo pigments, chronic exposure to ultraviolet light, and the role of additional factors, such as hepatitis C virus infection or interferon therapy (4).

The incidence, clinical symptoms, and severity of sarcoidosis vary according to race and sex, likely owing to the combined effects of genetic and environmental factors. In a study of Japanese patients with tattoo-associated sarcoidosis by Ohtsuka et al., features such as sex, age distribution, median tattoo duration, and frequency of intrathoracic lymphadenopathy were similar to those reported by Kluger in cases involving other racial groups. However, the frequency of uveitis is higher in Japanese patients with tattoo sarcoidosis than in patients of other ethnicities (5). This may reflect a genetic predisposition of Japanese individuals, as ocular involvement is more prevalent in Japanese patients with sarcoidosis than in Western populations. In previous reports, the treatment of tattoo sarcoidosis has frequently involved oral steroids, immunosuppressants, local treatment, and tattoo removal. However, there are also reports in which the condition naturally improved without treatment (6–11).

Although the etiology of tattoo sarcoidosis remains poorly understood, several mechanisms have been proposed. A key factor in the pathogenesis is the activation of the immune system, particularly a heightened Th1 immune response. Tattoo pigments are thought to act as exogenous antigens that trigger granulomatous reactions. This reaction may be exacerbated by the persistence of tattoo pigments in the dermis, which can serve as a continuous stimulus to the immune system even years after tattooing.

A theory suggests that immune reactions to tattoo pigments may trigger sarcoidosis, but there is also a theory that postulates that tattoos are not the cause, but rather a target for sarcoidosis. Tattoos may trigger an enhanced immune response in patients predisposed to sarcoidosis (5). Scar sarcoidosis is a condition in which skin sarcoidosis occurs in scars formed by trauma, surgical procedures, shingles, or other infections, and tattoos and permanent makeup can also be precursor conditions. Repeated skin microinjuries may also be involved in the development of Köbner's phenomenon (12, 13).

The etiology of sarcoidosis remains incompletely understood, although it is believed to arise from a complex interplay between genetic predisposition, environmental exposure, and immune dysregulation. Sarcoidosis is characterized by the formation of non-caseating granulomas that seem to result from an exaggerated immune response to unidentified antigens.

In Japan, the leading theory is that *P. acnes* is the causative bacterium of sarcoidosis. *P. acnes* DNA is frequently detected in sarcoidosis granulomas (14). PAB antibody is a monoclonal antibody that reacts with a glycolipid antigen called lipoteichoic acid distributed throughout the cell wall of *P. acnes* bacterial cell membranes, and when immunostaining is performed on sarcoidosis lesions, a large percentage of positivity is seen in the intracellular spaces of epithelioid cells and giant cells within the granulomas (15). *P. acnes* enters the body of healthy people via the respiratory tract, causing subclinical infection at the cellular level of the lungs and hilar lymph nodes, but an environmental factor or host predisposition may cause endogenous relapse in some people, resulting in chronic granuloma formation.

In the present case, *P. acnes* was detected within the granuloma of the skin lesion. To the best of our knowledge, this is the first case report of *P. acnes* detection in tattoo sarcoidosis. Whether this patient had a predisposition to sarcoidosis, whether tattooing exacerbated the condition, or whether the tattooing procedure caused *P. acnes*, which is normally present in the stratum corneum, to enter the subcutaneous tissue and cause sarcoidosis remained unknown. However, the fact that *P. acnes* was detected in the granuloma suggests that *P. acnes* may be involved in the pathogenesis of tattoo sarcoidosis. Until now, the suspected trigger for tattoo sarcoidosis was repeated microscopic damage to the pigment particles or to the skin itself, but we believe that the examination of increasing numbers of future cases for the presence of *P. acnes* will lead to a better understanding of the etiology of tattoo sarcoidosis.

In conclusion, this case of tattoo sarcoidosis highlights the potential role of *P. acnes* in the pathogenesis of this rare form of sarcoidosis. Detection of *P. acnes* within granulomatous lesions on tattooed skin suggests that this bacterium may play a role in the initiation or exacerbation of sarcoidosis in predisposed individuals.

## Data Availability

The raw data supporting the conclusions of this article will be made available by the authors, without undue reservation.
